# Enuresis in young offenders – a study on prevalence and mental health comorbidity

**DOI:** 10.3389/fpsyt.2024.1328767

**Published:** 2024-03-15

**Authors:** Roman A. Koposov, Andrew Stickley, Johan Isaksson, Vladislav Ruchkin

**Affiliations:** ^1^ Regional Centre for Child and Youth Mental Health and Child Welfare, Faculty of Health Sciences, UiT The Arctic University of Norway, Tromsø, Norway; ^2^ Department of Epidemiology and Modern Technologies of Vaccination, Institute of Professional Education, Sechenov First Moscow State Medical University, Moscow, Russia; ^3^ Department of Preventive Intervention for Psychiatric Disorders, National Institute of Mental Health, National Center of Neurology and Psychiatry, Kodaira, Japan; ^4^ Stockholm Center for Health and Social Change (SCOHOST), Södertörn University, Stockholm, Sweden; ^5^ Department of Medical Sciences, Child and Adolescent Psychiatry Unit, Uppsala University, Uppsala, Sweden; ^6^ Center of Neurodevelopmental Disorders, Centre for Psychiatry Research, Department of Women’s and Children’s Health, Karolinska Institute and Stockholm Health Care Services, Stockholm, Sweden; ^7^ Child Study Center, Yale University School of Medicine, New Haven, CT, United States; ^8^ Sala Forensic Psychiatric Clinic, Sala, Sweden

**Keywords:** enuresis, psychopathology, psychological problems, young offenders, mental health comorbidity

## Abstract

**Background:**

Enuresis is a common disorder in the school-age period, and is often associated with a variety of behavioral, psychological, and social problems. While early studies suggested an association between enuresis and delinquent behavior, there has been no recent research assessing the prevalence of enuresis and its comorbid psychopathology in young offenders. The aim of this study was to therefore assess the prevalence of enuresis and its associated psychiatric comorbidity in incarcerated young offenders.

**Methods:**

The prevalence of past and current enuresis and comorbid psychopathology was assessed using a semi-structured psychiatric interview and self-reports from 366 incarcerated male young offenders [age 14 to 19 years (mean age = 16.4)] from Northern Russia.

**Results:**

Seventy-three (20.0%) adolescents reported a previous history of enuresis, and in addition almost 10% of the youth reported current enuresis symptoms. Delinquent youth with enuresis did not significantly differ from other youth in the prevalence of comorbid psychiatric diagnoses when assessed by a clinical diagnostic interview, but had significantly higher levels of self-reported mental health problems, and suicidal ideation and attempts.

**Conclusion:**

Problems with enuresis are common among delinquent youth and may be associated with increased mental health problems. Given the potentially increased risk for suicidal thoughts and behavior in young offenders with enuresis, comprehensive mental health screening of those who are detected with this condition should be considered in the juvenile justice system.

## Introduction

Enuresis is a condition characterized by a recurrent involuntary micturition that occurs while sleeping (nocturnal enuresis), during the daytime (diurnal enuresis) or through a combination of both. Being one of the most common disorders in the school-age period, enuresis may also continue into adolescence and adulthood (1). It has been estimated that 10-15% of children at age 5 wet the bed during the night, with this rate decreasing from approximately 10% by the age of 7, to 5% by age 10 and to 1–3% in those age 18 and above (2–4). The overall prevalence of diurnal enuresis has been reported as being 8% in childhood and then decreasing to 0.5% in adolescence (5, 6).

Enuresis is associated with a variety of behavioral, psychological, and social problems (7–9) adding to children’s difficulties and the parental burden (10). Research has shown that multiple psychiatric problems are associated with enuresis (8, 11), with the reported prevalence of comorbid psychiatric disorders in some studies reaching as high as 89% (12). Research among children with enuresis suggests that in terms of comorbid conditions internalizing and especially externalizing problems are common (13–15), including attention-deficit/hyperactivity disorder (ADHD) (16), conduct disorder (CD) (17), tic disorders, anxiety, and obsessive-compulsive and oppositional-defiant disorders (8). Studies conducted among adolescents have similarly found that enuresis is associated with an increased risk of anxiety/withdrawal problems, as well as CD, ADHD symptoms (8, 9, 18) and suicidal behavior (19).

The association between enuresis and delinquent behavior attracted earlier academic attention in the period from the 1940s to the 1960s (20–23), where it was shown that enuresis was particularly common among delinquent adolescents, and that there was a close link between enuresis and “psychopathic traits”, defined as emotionally unstable traits similar to borderline personality, and persistent antisocial behavior (24, 25). However, research exploring enuresis and its associated problems in relation to delinquency has been generally limited to the last century (22, 26), with only a handful of more recent studies having been conducted (27). Indeed, to the best of our knowledge, there has been no research in recent years that has aimed to evaluate the prevalence of enuresis and its associated psychiatric comorbidity/mental health problems among delinquent adolescents. This may be an important oversight, considering previous reports have suggested that enuresis in delinquent youth, like many mental health problems (27), may be much more prevalent than in the general adolescent population.

Given this, the main goals of the present study were: 1) to estimate the prevalence of past and current enuresis symptoms in incarcerated young offenders; 2) to evaluate what types of psychiatric conditions might be associated with enuresis symptoms and 3) to compare self-reported mental health problems in delinquent youth with and without enuresis symptoms.

## Materials and methods

### Participants

#### Recruitment

Subjects (N=370) were recruited voluntarily, over a period of six months, from among the male adolescent inmates who had been sentenced to a period of incarceration in the only juvenile incarceration facility for males in the Arkhangelsk region of Northern Russia, a catchment area with a population of 1.5 million people, that is ethnically homogenous, with approximately 98% of the population being ethnically Slav.

This facility includes only male adolescents while female adolescents are placed in a different facility located in central Russia. Considering that there are substantially fewer adolescent girls who commit crime and are placed in correctional institutions when compared to boys, such facilities are few in number and generally include girls from different geographical areas.

The facility where the current study was conducted included the vast majority of criminally responsible male youths from the catchment area, who were placed in the institution by a court decision after committing a crime. The facility was not designed to specifically include youth with identified mental health problems. Those youth who are identified as having a severe mental health disorder after a forensic psychiatric examination and deemed in need of compulsory treatment are placed in a forensic psychiatric facility following a court decision. Adolescent offenders diagnosed with enuresis receive routine care from the medical staff affiliated to the facility and they have the possibility of receiving clean sheets. They can also receive psychological support from psychologists working at the facility.

The age of criminal responsibility in Russia is generally 16 years, but for the most severe crimes, criminal liability begins at the age of 14. In the case of longer sentences juveniles can be transferred to an adult facility after having reached 18 years of age but may be allowed to stay in a juvenile facility until the age of 19 years. The rights of incarcerated adolescents are protected by the Commissioner for children’s rights under the Governor of the Arkhangelsk region.

Most of the detainees had multiple convictions that included property crimes (theft 51%), violence-related crimes (e.g. fighting, robbery 38%), and in some cases rape/sexual violence (6%) or murder (5%). Generally, those institutionalized for theft had shown a repetitive pattern of stealing, with multiple convictions, who were usually sentenced to the facility following a repeat conviction during parole. The mean sentence length at the time of the study was 4.3 years; while all of the participants had been incarcerated (including the detention period) for at least 6 months at the time of the study.

#### Ethical approval

This research was approved by the ethical committee at the Northern State Medical University in Arkhangelsk (Russia). All potential participants were given detailed descriptive information about the study and were informed about the voluntary and confidential nature of their participation. They were further assured that the staff at the facility would not obtain any individual information about their responses. Informed consent was obtained from all participants and in addition, also from the director of the juvenile correctional facility.

### Measures

Psychopathology was assessed using a semi-structured psychiatric interview, which was conducted with 370 delinquent youths. During the course of the study some youths were released, whereas others entered the facility, thus the interviewed group appears to be larger than the total population of the institution (N=300) at any particular time. The psychiatric assessment was conducted individually; self-report data were obtained during small group sessions (5-8 participants).

The *Schedule for Affective Disorders and Schizophrenia for School-Age Children-Present and Lifetime Version* (K-SADS-PL), a commonly used semi-structured psychiatric interview with high inter-rater diagnostic reliability and extensive validation work (28), was used to yield current and past psychiatric diagnoses. To assign a diagnosis, clinically significant diagnosis-specific impairment had to be present. Interviews were conducted by two experienced psychiatrists, who determined the presence of diagnoses using DSM-IV criteria (29). Interrater reliability for this measure is high, with interrater agreement in scoring screens and diagnoses ranging from 94% to 100% (28). As the purpose of the present study was to assess the relationship between a lifetime history of enuresis and psychopathology, the lifetime rates of psychiatric diagnoses were used in the analyses. In addition to the psychiatric diagnoses, we used data on previous suicidal ideation and attempts, which was obtained using the Depressive Disorders module of the K-SADS-PL.

The assessment of enuresis symptoms is included in the screening interview of the K-SADS-PL and inquires about repeated voiding at night and during the daytime, currently and in the past, with the cut-off level of a minimum of one to four times a month for three or more months. For the purposes of the present study, we combined those with nocturnal and diurnal enuresis symptoms into one group, given that a previous report found that they do not differ in terms of associated psychopathology (30), but included a separate assessment of symptoms in the past and in the present. Thus, the participants were pooled in three groups: 1) no enuresis, 2) with a past history of enuresis only and 3) with current symptoms.

The *UCLA Post-Traumatic Stress Disorder Reaction Index* for children and adolescents (PTSD-RI) is a self-report instrument designed to assess posttraumatic stress experiences and symptoms among children and adolescents. In this study, a section assessing how frequently 20 posttraumatic stress symptoms occurred during the past month was used. This instrument is one of the most commonly used measures of PTSD in childhood, and has been widely used internationally (31). Cronbach’s alpha for the scale was 0.81 in the present sample.

The *Beck Depression Inventory* (BDI) (32) consists of 20 items with four response alternatives (ranging from 0 to 3) describing individual feelings and behaviors related to depression, with higher scores indicating more depressive symptoms. Earlier research has shown that the BDI has acceptable psychometric properties in both psychiatric and non-psychiatric samples, both internationally (32) and among Russian adolescents (33). Cronbach’s alpha for the scale in the present sample was 0.81.

Information was also obtained using the *Youth Self-Report* (YSR) (34), which was designed to obtain youths’ view of their competencies, feelings and behavioral/emotional problems in a variety of areas, including, being withdrawn, somatic complaints and anxious/depressed problems (internalizing problem scale), delinquent and aggressive behaviors (externalizing problem scale), and social, thought and attention problems. The YSR contains 112 items, which are rated 0 (not true), 1 (somewhat or sometimes true), and 2 (very true or often true). A total score can be computed both for the internalizing and externalizing scale, with higher scores indicating higher levels of behavioral and emotional problems. The scale has been previously validated in Russia (35) and used with young offenders (36). In the present sample Cronbach’s alpha was 0.89 for the internalizing scale and 0.87 for the externalizing scale.

The translation of these scales into Russian followed established guidelines, including the use of independent back translations (37). The Russian translation of the K-SADS-PL was done at the Department of Psychology, Moscow State University. After the translation was completed, the instruments were reviewed by native Russian-speaking mental health colleagues. An official interpreter made independent back translations. The obtained versions were reconciled, analyzing and correcting inconsistencies.

### Statistical analysis

The data were analyzed using SPSS, version 25. Chi-square tests were used to assess differences in psychiatric comorbid disorders, and one-way ANOVA tests were utilized when comparing self-reported mental health problems between the three groups of adolescents (with past, current and no enuresis). *Post-hoc* Bonferroni corrections were applied to evaluate the differences in self-reported problem scores between 1) those with past enuresis symptoms only and those without enuresis symptoms, 2) those with current enuresis symptoms and those without enuresis symptoms.

As some of the adolescents who participated in the psychiatric interview did not also complete self-reports, the reported number of individuals for the one-way ANOVA tests was smaller than that for the Chi-square tests.

## Results

Among the 378 recruited subjects, eight refused to participate because of an unwillingness to provide the detailed personal information that was inquired about during the course of the psychiatric interview. In addition, four cases were subsequently excluded because of missing data, hence 366 participants were included in the analysis. The age of the participants ranged from 14 to 19 years (mean age = 16.4, sd = 0.9).

### Prevalence of enuresis

Of the 366 participating subjects, 73 (20.0%) described a previous history of enuresis but denied any current enuresis symptoms at the time of the study, while an additional 36 (9.8%) participants reported current symptoms. Of those with current symptoms, 28 (77.8%) also described a previous history of enuresis.

### Psychopathology

**Table 1** shows the prevalence of lifetime comorbid psychiatric diagnoses in delinquents with and without past/current symptoms of enuresis. Both those with a past history of enuresis and those with current symptoms did not differ significantly from those with no enuresis on any of the lifetime psychiatric diagnoses.

**Table 1:** Prevalence of lifetime psychiatric diagnoses in young offenders with and without enuresis [N (%)].

Other diagnosis	No enuresis (N=257)	Past enuresis (N=73)	Current enuresis (N=36)	Chi-square p ^e^
MDD ^a^	25 (9.7)	12 (16.4)	5 (13.9)	3.75 (.253)
Mania	25 (9.7)	9 (12.3)	4 (11.1)	0.44 (.804)
Anxiety Disorder	35 (13.6)	13 (17.8)	7 (19.4)	1.39 (.499)
PTSD ^b^	56 (21.8)	20 (27.4)	10 (27.8)	1.40 (.496)
CD ^c^	190 (73.9)	57 (78.1)	22 (61.1)	3.65 (.161)
ADHD ^d^	40 (15.6)	15 (20.5)	10 (27.8)	3.71 (.156)
Alcohol Abuse	143 (55.6)	42 (57.5)	21 (58.3)	0.15 (.927)
Other Substance Abuse	70 (27.2)	14 (19.2)	9 (25.0)	2.52 (.113)
Any Psychiatric Diagnosis:				
1-2	137 (53.3)	41 (56.2)	12 (33.3)	7.71 (.103)
3 or more	91 (35.4)	28 (38.4)	18 (50.0)	

a MDD, Major depressive disorder.

b PTSD, Posttraumatic stress disorder.

c CD, Conduct disorder.

d ADHD, Attention-deficit/hyperactivity disorder.

e p, p-value.

With regard to suicidal ideation (data not shown in the table), among those with either past or current enuresis symptoms 28.7% reported recurrent thoughts of death as compared to 15.6% in the group without enuresis symptoms (Chi-square=10.32; p<.01). In addition, 23.1% of those with either past or current enuresis symptoms reported frequent suicidal ideation and had thought of a specific method for suicide, as compared to 12.1% in those without enuresis symptoms (Chi-square=10.72; p<.01). Finally, as regards the seriousness of previous suicide attempts, 17.6% of those participants with either past or current enuresis symptoms reported an intent to die during their previous suicide attempt, as compared to 9.7% in those without enuresis symptoms (Chi-square=17.98; p<.001).

The mean scores for different problems as assessed by the YSR, BDI, and PTSD-RI self-report scales are presented in **Table 2**. Those with past or current enuresis symptoms generally had higher problem scores, as compared to those without enuresis symptoms. Those with current symptoms did not differ from those with only past enuresis symptoms on any of the problem scales. Those with current (but not those with past) enuresis symptoms scored higher than those without enuresis symptoms on the PTSD-RI, and YSR social problems and thought problems. Those with past (but not current) enuresis symptoms scored higher than those without enuresis symptoms for the YSR somatic symptoms and attention problems, and on the BDI. Finally, those with past or current enuresis symptoms also scored higher than those without enuresis symptoms on the YSR withdrawn, anxious/depressed and self-destructive behavior categories and also had a higher internalizing problems total score.

**Table 2:** Results of a one-way ANOVA ^g^ comparing problem scores in young offenders in relation to enuresis (M ^h^ [SD ^i^)].

Variables	No enuresis (N=249)	Past enuresis (N=66)	Current enuresis (N=31)	Statistics F ^j^, p ^k^
PTSD-RI ^c^	24.44 (12.40)	28.17 (13.09)	31.63 (11.39)	5.63, <.01 ^b^
BDI ^d^	16.64 (11.45)	21.00 (10.64)	20.10 (13.42)	4.31, <.05 ^a^
YSR ^e^:				
Withdrawn	4.69 (2.54)	5.76 (2.84)	6.40 (2.99)	7.97, <.001 ^a,b^
Somatic Complaints	3.86 (3.10)	5.48 (3.80)	4.23 (3.52)	5.57, <.01 ^a^
Anxious/Depressed	9.12 (6.25)	12.02 (5.81)	12.43 (6.61)	7.59, <.001 ^a,b^
Social Problems	5.56 (2.41)	5.41 (2.98)	6.07 (2.89)	6.12, <.01 ^b^
Thought Problems	3.79 (2.97)	4.45 (3.10)	5.80 (2.72)	6.46, <.01 ^b^
Attention Problems	6.81 (3.20)	8.26 (2.91)	8.03 (3.38)	5.95, <.01 ^a^
Delinquent Behavior	8.07 (3.75)	8.90 (4.11)	8.43 (4.06)	1.08, ns ^f^
Aggressive Behavior	12.82 (6.48)	13.72 (6.52)	14.03 (7.75)	0.74, ns ^f^
Self-Destructive Behavior	4.94 (4.09)	6.52 (3.94)	6.90 (4.21)	5.62, <.01 ^a,b^
Internalizing Problems	17.13 (9.84)	22.39 (10.31)	22.33 (10.97)	8.44, <.001 ^a,b^
Externalizing Problems	20.64 (9.34)	22.40 (9.91)	22.43 (11.20)	1.06, ns ^f^

Post-hoc Bonferroni tests showing significant differences between:

^a^ - those with past enuresis symptoms only and those without enuresis symptoms,

^b^ - those with current enuresis symptoms and those without enuresis symptoms.

^c^PTSD-RI, Post-traumatic stress disorder reaction index.

^d^BDI, The Beck depression inventory.

^e^YSR, Youth self-report.

^f^ns, Non-significant.

^g^ANOVA, Analysis of variance.

^h^M, Mean value.

^i^SD, Standard deviation.

^j^F, F-value.

^k^p, p-value.

## Discussion

To the best of our knowledge, this is the first study in recent years to use a structured diagnostic assessment to investigate the prevalence of enuresis symptoms and their associated psychiatric comorbidity in incarcerated young offenders. The study also aimed to assess whether delinquent youth with and without enuresis symptoms differed on self-reported mental health problems.

It was found that every fifth participant reported a previous history of enuresis symptoms, and in addition almost 10% of youth had current enuresis symptoms. These figures are higher than those reported among adolescents from the general population (4), suggesting that delinquent adolescents may represent an at-risk group for this disorder, hence potentially supporting the previously described association between enuresis and CD (18).

Somewhat unexpectedly, when using a clinical diagnostic interview, no significant differences were found in the prevalence of comorbid psychiatric diagnoses between delinquent youth with and without enuresis symptoms. This finding contrasts with the results from older, descriptive studies undertaken among delinquent adolescents in the middle of the last century, which reported a link between enuresis and mental health problems (24, 25). At the same time, while there is some evidence that the rates of psychiatric disorders in children with enuresis may be higher than in non-enuretic groups (38), most children with enuresis demonstrate no symptoms of emotional or behavioral disturbance (39). The current findings are further supported by the limited psychosocial impairment in children with enuresis (39), which seems to be mostly associated with various degrees of psychosocial stress and damage to self-esteem in affected children (40).

When assessed by self-reports, however, differences were found for several problem scores between those with either past or current enuresis symptoms and those without enuresis, but not between those with current and past enuresis symptoms. Adolescents with current enuresis symptoms reported higher levels of posttraumatic stress, social and thought problems than those without enuresis. Delinquent youth with past enuresis symptoms had more somatic symptoms, attention problems and were more depressed than those without enuresis symptoms. Similar to the results from the clinical interview, adolescents with and without enuresis did not differ in terms of either aggressive or delinquent behaviors, which does not concur with the results from previous research (8). This contradictory finding may be potentially explained by the fact that the study was conducted on a population of incarcerated youth with documented problems with delinquent behavior, and with supposedly lower levels of variation in externalizing problems, as compared to adolescents in the general population, such as those described in a study by Joinson et al. (15), where children from the general population with combined bedwetting were particularly at risk for externalizing problems. Thus, our results suggest that adolescents with varying degrees of enuresis symptoms, may also have an increased level of mental health problems, which, however, does not reach the level of being clinically diagnoseable. These findings are seemingly supported by the results from other studies, such as by Biederman and coauthors (30), where enuresis did not increase the risk for psychopathology in children with or without ADHD and was not associated with psychosocial adversity. Hence, although enuresis has often been considered as an expression of psychological disturbance among children, it may be difficult to determine whether the relationship between enuresis and mental health problems is of etiologic relevance or has occurred in response to the distressing symptoms of enuresis (39).

The finding that a higher proportion of adolescents with enuresis also report more frequent suicidal ideation and attempts, than those without enuresis symptoms, may be also potentially associated with the increased levels of psychosocial stress that can arise from the condition and its associated problems. For example, enuresis is often a cause of shame and stigma, and among youth may lead to teasing and bullying, especially in an institutional environment, where it may be virtually impossible to hide such problems from other youth. Being stressed and bullied might in turn, result in subsequent suicidal thoughts and behaviors (41–43). However, in our study we did not assess either emotional stress or bullying and thus can only hypothetically assume that they may have a role in the association between enuresis and suicidal behavior. Although, we did not find any evidence that there is increased psychiatric comorbidity at a diagnostic level in adolescents with enuresis, earlier research suggests that there may be an increased risk for some psychiatric disorders, including ADHD and PTSD (8, 9), which indirectly is supported by the higher scores that were obtained by the participants for posttraumatic stress, and attention problems and anxiety/withdrawal scores on the YSR in the present study. At the same time, there is some inconsistency in the findings from previous research regarding ADHD as not all studies have found increased comorbidity between enuresis and ADHD (30).

### Strengths and limitations

The strength of this study relates to the fact that we were able to use structured assessment methods while examining the association between enuresis and comorbidity in delinquent adolescents. Nonetheless, there are several limitations that need to be considered. As the study sample comprised Russian young offenders placed in a correctional facility in only one location the generalizability of these results to either other parts of Russia or elsewhere is uncertain. Also, while the assessment of adolescents was conducted by means of both clinical interviews and by self-reports, socially desirable responding might have been an issue. Other limitations relate to the fact that the study included only males and might have been underpowered to detect differences in psychiatric diagnoses. In addition, being able to add parental reports to confirm the data reported by adolescents would have improved confidence in the findings but this was not possible to do in the framework of the current study.

## Conclusion

The presence of comorbid psychological and behavioral problems in delinquent adolescents with enuresis together with higher levels of suicidal ideation and more suicide attempts has potential implications for treatment, as unrecognized psychological problems may lead to poorer outcomes and treatment failure (44). The treatment of enuresis at this age can be particularly challenging due to a low compliance with therapy (44, 45), which might be due, at least in part, to the shame and stigma associated with enuresis (46). Indeed, worrying about being bullied and teased threaten the psychosocial development of young people (10) and may lead to an unwillingness to be diagnosed at all. It can be speculated that this might be especially the case for incarcerated adolescents considering the specificity of their subculture and certain hierarchy of relationships (47). Regardless, the results of this study highlight the importance of identifying enuresis in incarcerated adolescents, screening them for comorbidity and then undertaking therapy to minimize the associated burden and risk of continuing this disorder to adulthood.

## Data availability statement

The raw data supporting the conclusions of this article will be made available by the authors, without undue reservation.

## Ethics statement

The studies involving humans were approved by the ethical committee at the Northern State Medical University in Arkhangelsk (Russia). The studies were conducted in accordance with the local legislation and institutional requirements. Written informed consent for participation in this study was provided by the participants’ legal guardians.

## Author contributions

RK: Writing – original draft, Writing – review & editing, Conceptualization, Methodology. AS: Conceptualization, Methodology, Writing – review & editing. JI: Conceptualization, Methodology, Writing – review & editing. VR: Conceptualization, Data curation, Methodology, Writing – original draft, Writing – review & editing.
